# Cancer vaccines at an inflexion point: what next?

**DOI:** 10.1186/1479-5876-9-148

**Published:** 2011-09-09

**Authors:** Adrian Bot, Mihail Obrocea, Francesco M Marincola

**Affiliations:** 1 Kite Pharma, Inc., Los Angeles CA 90024, USA; 2 Global Pharmaceutical Research and Development, Abbott Laboratories, Redwood City, CA 94063, USA; 3 Infectious Disease and Immunogenetics Section (IDIS) - Department of Transfusion Medicine, Clinical Center, and trans-NIH Center for Human Immunology (CHI) National Institutes of Health, Bethesda, MD 20892, USA

## Abstract

With the approval of the first therapeutic cancer vaccines for veterinarian and human use, the field reached a significant milestone after a considerable interval of tumultuous research and development marked by numerous ups and downs. As the mechanism of action and clinical benefit afforded by this class of agents are starkly different from that of conventional or small targeted therapies for cancer, there are still numerous hurdles that need to be overcome to fully unleash their potential. These challenges and efforts are illustrated in a book just published on this subject, a non-exhaustive yet representative synopsis of the latest advances in cancer vaccine technologies in various stages of development. Major lessons resulting from clinical testing of cancer vaccines and other immune interventions, are being integrated in novel, cutting edge platform technologies that blur the distinction between passive and active immunotherapies as well as carry the promise of fundamentally changing and improving the management of patients with cancer.

## 

It took more than a century after the first published description of cancer treatment through active immunotherapy by Coley [1], half a century since the formulation of the "cancer immunosurveillance" hypothesis by Burnet [2], and several decades since the discovery of dendritic cells by Steinman [3], MHC class I-restricted cytotoxic lymphocytes by Zinkernagel and Doherty [4] or tumor associated-antigens by Boon and Rosenberg [5,6], to finally witness the arrival of the first commercial veterinarian [7] and human [8] antigen-targeted therapeutic cancer vaccines.

The mere subject of cancer vaccines could still trigger hot debates, finding its fervent opponents or its staunch supporters in the scientific and clinical community. As the arguments of the former are firmly anchored in the belief that the immune system has essentially evolved to ensure anti-microbial as opposed to anti-tumor monitoring, the latter segment of the scientific community sees the appealing and imminent possibility of translating the immunity's major features to groundbreaking therapies.

The skepticism around immune interventions and in particular cancer vaccines had objective roots as key questions and hurdles needed to be tackled to fully unleash their potential in clinic: a) their indirect mechanism of action manifests through a rather subtle and sometimes delayed clinical benefit; b) the latter could be evident in most cases at cohort rather than patient's individual level; c) there are very few instances of objective, durable responses owing to poorly understood mechanistic circumstances; and d) the reliability of current immune correlates of clinical benefit was questionable despite significant progress in immunology. All these issues translated into an exceedingly difficult, risky and expensive process of developing such therapeutic agents. The decision-making for optimization, advancement to next stage or termination has been essentially deferred to large phase 2 or 3 clinical trials, unless the industrial sponsor succumbed to inherent challenges of a competitive market economy. The proponents of therapeutic cancer vaccines, in turn, advocated the need to rationally and systematically tackle the general challenges from above. However, vaccine technologies also present specific challenges: autologous cell based vaccines have expensive and cumbersome manufacturing processes with economical and commercial implications, while "off the shelf" vaccine platform technologies have yet to show clinical benefit.

Marking the arrival of the first antigen-targeted cancer vaccines on the market, the book published this month [9] aims to also provide the readership with a perspective across key platform technologies in development, challenges and hurdles ahead as well as strategies to overcome those barriers. In addition to cell based and microbial vaccines, a new generation of potent synthetic vaccine candidates facilitated by the discovery of Toll-like receptors [10] emerged, with more favorable economics associated with their development and optimization, carrying the promise of reducing the optimization cycle and propelling this field towards other regulatory approvals. Along with efforts to improve on vaccines' intrinsic potency, much of the current focus is dedicated to advancing reliable predictive biomarkers and immune monitoring resulting from a rationale systems-biology approach. These three key areas of research aim to maximize the potential of cancer vaccines through their unique mechanism of action, in the quest to generate durable responses and tackle disseminated disease.

Nevertheless, in this darwinistic environment representing today's "state of affairs" in cancer therapeutics development, platform technologies cross-communicate with potentially astonishing outcomes. This is reflected by new trends and emerging technologies [9], essentially obscuring the distinction between passive and active immunotherapies. From efforts directed at whole immune system reengineering through T cell programming and adoptive cell transfer therapies, we should also expect key learning with a potential to translate to very innovative and "off-the-shelf", widely applicable immune interventions. As this phenomenon of cross-fertilization between different platform technologies evolves in front of our eyes, a natural question comes to our mind: could the concept of "therapeutic cancer vaccination" survive as such or rather evolve past its original definition, in this rapidly changing landscape?
